# Retransmission Avoidance for Reliable Data Delivery in Underwater WSNs

**DOI:** 10.3390/s18010149

**Published:** 2018-01-07

**Authors:** Babar Ali, Arshad Sher, Nadeem Javaid, Saif ul Islam, Khursheed Aurangzeb, Syed Irtaza Haider

**Affiliations:** 1 COMSATS Institute of Information Technology, Islamabad 44000, Pakistan; babar93.282@gmail.com (B.A.); arshadsher92@gmail.com (A.S.); saiflu2004@gmail.com (S.u.I.); 2 College of Computer and Information Sciences, King Saud University, Riyadh 11543, Saudi Arabia; kaurangzeb@ksu.edu.sa (K.A.); sirtaza@ksu.edu.sa (S.I.H.)

**Keywords:** re-transmissions, energy efficiency, energy hole, multipath, layered path, embedded systems, feasible regions, cross node, reliable data delivery

## Abstract

The energy-efficient and reliable delivery of data packets in resource constraint underwater wireless sensor networks (UWSNs) is one of the key considerations to enhance the network lifetime. The traditional re-transmissions approach consumes the node battery and increases the communication overhead, which results in congestion and affects the reliable data packet delivery in the network. To ensure the reliability and conserve the node battery, in this paper, we propose adaptive forwarding layer multipath power control routing protocol to reduce the energy dissipation, achieve the data reliability and avoid the energy hole problem. In order to achieve the reliability, tree based topology is exploited to direct multiple copies of the data packet towards the surface through cross nodes in the network. The energy dissipation is reduced by a substantial amount with the selection of low noise path between the source and the destination including the information of neighbors of the potential forwarder node. Extensive simulation results show that our proposed work outperforms the compared existing scheme in terms of energy efficiency and packet received ratio (PRR).

## 1. Introduction

Recent progress in underwater wireless sensor networks (UWSNs) has procured much attention and it encompasses an extensive range of applications like pollution monitoring, coastline surveillance, disaster prevention, oceanographic data collection, marine life monitoring, etc. [1]. UWSN is composed of sensor nodes deployed in the deep water which forward data to sink(s) situated off shore. Radio and optical waves used in terrestrial communication cannot be used in underwater environment because they get absorbed or scattered rapidly. Therefore, the acoustic signals emerge as a suitable choice due to low absorption rate.

Acoustic signals face high end-to-end delay because of low propagation speed, whereas, multipath fading, doppler effect, path loss, noises like shipping, wind, thermal, etc. cause high bit error rate (*BER*) resulting in low reliability and high energy consumption [2]. Acoustic signals are stronger near the source and get impaired from noises as they propagate away. Noise intensity in shallow water is high as compared to deep water. Therefore, less number of different paths are required in deep water to reduce the energy consumption. Sensor nodes are costly and equipped with limited batteries which are difficult to replace. Therefore, energy efficiency, maximum data gathering, reliable transfer and low delay are the objectives of protocol designing.

Some multipath routing protocols [3,4] have been proposed in recent years to achieve reliability. Data packets are forwarded linearly through different paths towards the sink(s) situated at the surface of the water. If these copies, which are less in number, somehow survived energy holes, there are high noises in shallow water which make them erroneous resulting in data packet drop at the sink and high energy consumption. Data packets are also forwarded by following tree structure in which every sensor node multicasts the data packet upon reception in its transmission range and becomes the parent while, the receivers further multicast data packet and make subtrees. The reception of multiple copies at the sink increases the chances of uncorrupted data packet reception. However, a high number of copies increases collision and energy consumption which is unbearable with the availability of limited node battery.

Authors of layered multipath power control (LMPC) [5] send multiple copies of a data packet by following binary tree structure packet. These copies are directed through different paths which faces low noise to achieve reliability. They are combined at the surface to generate original data packets. LMPC generates binary tree from every source node resulting in high energy utilization in dense network. Sparse regions created by random deployment or death of sensor nodes result in data packet drop because of void holes (unsensed zone) in LMPC.

In this paper, we propose forward layered multipath power control-one (FLMPC-One) and FLMPC-Two routing protocols to forward the data packets towards the surface through multiple paths with the aim of eliminating retransmissions and avoiding void holes. Retransmission increases delay and energy consumption which effects the performance of time critical scenarios. In order to reduce the energy dissipation, the network field is divided into multiple heterogenous sized layers. From each layer, the source node unicasts the data packet which resides near the layer, whereas, node on the layer known as cross node generates binary tree upon reception of the data packet by using IP multicast technology. Current forwarder in FLMPC-One forwards data when it is confident about finding neighbors up till two hops. While, FLMPC-Two uses three hops neighbor information. The goal of FLMPC-One and FLMPC-Two is to achieve energy efficiency and reliability of the data packet.

The main contributions are mentioned as follow:

- Binary tree establishment from the sensor node lies on the layer nearest to the source node which results in less energy consumption.
- In order to avoid void holes, the forwarder node sends data packet to that sensor node which further has one or two hop neighbor nodes in its transmission range.
- Every data packet passing through sensor nodes lies on the layer which reduces the chance of retransmissions due to the reception of more than one copies at the sink.

The remainder of the paper is organized as follows: Section 2 provides an overview of existing routing protocols in UWSNs. In Section 3, a brief overview of FLMPC-One and FLMPC-Two is provided along with problem description in LMPC. Linear optimization of energy and throughput are discussed in Section 4. Section 5 consists of simulation results and performance evaluation of both proposed schemes and LMPC. In Section 5.3, performance trade offs are discussed. Finally, Section 6 presents conclusion.

## 2. Related Work

In this section, we describe recently proposed schemes which achieve energy efficiency and high throughput. For better understanding and in-depth knowledge, depth based state of the art is categorized in to: multi-hopping through single path to achieve minimal energy consumption and avoid void hole, whereas, multi-hopping via multiple-paths is performed to ensure the reliable data delivery at the destination. The brief discussion is provided in the following subsections.

### 2.1. Depth Based Routing Using Single Path

Yan et al. proposed depth based routing (DBR) for UWSNs in [6]. DBR forwards the data packet greedily to the lowest depth node in transmission range using local information of one hop. Every sensor node updates its own depth in each data packet while forwarding. Therefore, the higher depth nodes discard it upon reception which helps to avoid redundant data packets. In order to reduce duplication, every node maintains queue which stores information of the forwarded data packets. The sensor nodes require more memory to maintain queues. DBR also uses data packet holding mechanism. Lowest depth node in the transmission range holds data packet for shortest time. DBR performs well in terms of packet delivery ratio (PDR) and delay in dense regions, however, sparse regions affect them because of void holes in the network.

In [7], Yu et al. proposed weighting depth and forwarding area division depth based routing (WDFAD-DBR) protocol for underwater acoustic sensor networks (UASNs). The authors use weighting sum of depth difference of current and next expected hop technique to improve reliability and reduces probability of void hole occurrence. WDFAD-DBR saves energy by dividing forwarding area to suppress redundant data packets and neighbor node prediction mechanism which reduces the chance of packet drop. Authors performed theoretical analysis on routing performance in case of channel contention. WDFAD-DBR increases reliability and achieves energy efficiency. However, sparse region affects PDR and processing time at each hop due to the selection of forwarder increases delay.

The authors proposed a reliable and energy-efficient routing (R-ER$P^{2}$R) protocol for UWSNs in [8]. This protocol balances energy consumption and decreases delay by utilizing physical distance at the time of a data packet transmission. Every sensor node calculates expected transmission count for each of its neighbor to find the link quality for reliable transmission of the data packets. A lowest depth neighbor in the range with high residual energy and good link quality is selected as forwarder. R-ER$P^{2}$R forwards only one copy of a data packet to increase network lifetime. However, if a data packet reaches void region, sensor node drops it which results in retransmissions.

In [9], Noh et al. proposed void-aware pressure routing (VAPR) for UWSNs. VAPR works in enhanced beaconing and opportunistic directional data forwarding phases. All the sensor nodes forward data packets to the sink on the basis of pressure routing which uses depth information. The mobile sinks propagate their reachability information and the sensor nodes update their distance to sink from this control message. Afterwards, the sensor nodes beacon their information which helps the neighbors to update direction of forwarding and hop counts to sink. Hop count information in the beacon messages helps the sensor nodes to avoid void regions. VAPR performs better in dense regions. However, sparse regions affect its performance in terms of delay and PDR.

Improving both energy and time efficiency of depth-based routing (D-DBR) for UWSNs is proposed by Diao in [10]. This protocol uses time of arrival (ToA) ranging technique to measure distance between sensor nodes. Every sensor node makes decision of forwarding the received data packet based upon distance, depth and transmission range information. In order to direct data packets towards the sink, every sensor node calculates the angle between the sink and all the neighbors of the current forwarder which helps to select potential forwarder. D-DBR reduces delay and increases PDR of the network by directing packets into right direction at the cost of energy.

Jiang et al. [11] proposed geographic multipath routing based on geospatial division in duty-cycled UWSNs. Two schemes named; geographic forwarding based on geospatial division (GFGD) and greedy GFGD (GGFGD) are proposed in this research work. The sensor nodes can switch their state among sleep and awake modes which saves energy of idle sensing. Neighbor node having high residual energy, shortest transmission delay and less path loss is selected as a forwarder in both schemes. GGFGD greedily forwards data packet and selects next hop node closer to the sink. While, GFGD forwards data packet directionally. GFGD saves more energy and have less delay as compared to GGFGD. Farthest neighbor selection in GGFGD increases propagation delay. Summary of the related work is given in Table 1.

### 2.2. Depth based Routing using Multiple Paths

Multipath power-control transmission (MPT) protocol for time critical applications is proposed in [12]. Every source node directs multiple copies of data packet towards the surface gateways with the purpose of avoiding retransmissions. These copies are then combined at sink to get original data packet. MPT performs well in terms of delay and energy consumption in sparse regions. However, as the node density increases both delay and energy consumption increase because of data packets collision in the network.

Javaid et al. proposed an efficient data-gathering routing (AEDG) protocol for UWSNs in [13]. This protocol uses autonomous underwater vehicle (AUV) to collect data from randomly deployed gateway nodes. During attachment to gateways, every sensor node follows shortest path tree algorithm. Gateway node collects data from member nodes and makes sure that less number of nodes are attached to it, because the involvement of more number of nodes resulting in high energy consumption. AEDG rotates gateway nodes in order to balance energy consumption. New gateway node is selected on the basis of high residual energy and it must be in the range of AUV. AEDG prolongs network lifetime and increases throughput. However, end-to-end delay is compromised.

A power-efficient routing (PER) protocol for UWSNs which uses forwarding node selector mechanism to find appropriate forwarder is proposed in [14]. Every sensor node generates binary tree to direct multiple copies of a data packet to sink. In order to control duplication, every node uses forwarding tree trimming mechanism. If the number of duplicate packets forwarded by a sensor node exceeds threshold, the sensor node drops it and trims the tree. This protocol uses fuzzy logic inference system and decision tree which help to select appropriate forwarder and generate the tree. PER increases PDR at the cost of delay and energy. Duplicate data packets information maintenance at every node consumes a lot of energy.

Xu et al. proposed reliable and energy-efficient multipath communications in UWSNs [15]. In this protocol, multiple path forward error correction (M-FEC) based on hamming code technique is used. This technique encodes data packet at source node which is decoded at every intermediate node and the destination in order to reliably transmit data packet in the network. To calculate packet error rate (*PER*), authors use markovian model. Multiple copies of a data packet are sent through different paths by source node to avoid retransmissions. The source node adds error correction code in each data packet before transmission which allows the receiver to detect and correct errors. When a data packet reaches the destination, all the copies are combined to generate original data packet. M-FEC achieves reliability and saves energy by eliminating retransmissions. However, data packet correction at each node increases delay.

Energy balanced strategies for maximizing the lifetime of sparsely deployed UASNs are proposed in [16]. In this research work, authors proposed energy balanced hybrid (EBH) data propagation algorithm which efficiently utilizes energy of the network. All the sensor nodes can switch their state among direct transmission and multi-hopping which reduces packet drop. Initially, the sensor nodes send data packets through multiple hops towards the destination. When a node’s energy depletes, it propagates control message to the higher depth nodes. These high depth nodes start direct transmission with the sink node. EBH balances energy consumption, however, direct transmission and void holes increase energy consumption and delay. The limitations and achievements are summed up in Table 2.

### 2.3. Multi-Hopping to Avoid Void Regions

Authors proposed in [17], the geographic and opportunistic routing protocol (GEDAR) for UWSNs. This is an anycast, geographic and opportunistic protocol which selects a potential neighbor to forward data packet using anycast method. Every sensor node greedily forward data packets to the lowest depth node in its range. When a data packet reaches void region, the current forwarder switches to recovery mode and changes depth to find a potential neighbor. GEDAR overcomes void holes and increases PDR in dense regions. However, position changing mechanism consumes more energy and increases delay.

In [18], the authors proposed hydrocast: pressure routing for UWSNs which uses measured pressure level information to route data packets towards the destination. Every data packet is routed by anycast mechanism in only vertical direction. Every sensor node forwards each data packet to a subset of forwarders which maximizes the greedy progress towards the destination. The receiver node does not forward data packet until all the higher priority nodes in forwarding set fails. If a data packet reaches in void region, the current forwarder sends it to a higher depth node (backward transmission) and directs it to the sink through another path. The reliability increases by sending data packet to multiple forwarders and sends using different path in case of void region. However, hydrocast pays the cost of energy and delay.

The authors of on energy hole and coverage hole avoidance in UWSNs proposed spherical hole repair technique (SHORT) in [19]. Coverage hole is un-sensed region which occurs because of uneven distribution of nodes, random deployment or by death of sensor nodes. Every sensor node embeds its residual energy information in the forwarding data packet which helps the receivers to update their neighbors information. In order to reduce packet loss and suppress retransmissions, holding time is computed for each packet before forwarding. When a sensor node is near to die, it generates control message to its neighbors so, they stop forwarding data to it. Now a sensor node in dense region moves to fill this coverage hole ensuring its movement does not create another coverage hole. The nodes which make cross triangles or hidden cross triangles have higher probability of movement. Coverage hole filling increases throughput and lifetime of the network at the cost of high delay. The summary of the discussed routing algorithms is provided in Table 3.

### 2.4. Summary of Related Work

In aforesaid state of the art, the major focus of the research community is on the energy savings through multi-hop based routing. However, the features of the existing work differs from each other as per the requirements of the scenario. Such as, depth routing algorithms with single path focus on energy efficiency by ignoring the inevitable threat of void holes results in discontinuity in the network operations.

In multipath depth based routing schemes, source node looks for the reliability of the data packet. The scarcity of the energy is not taken into the consideration and packets are transmitted from various paths towards the destination resulting in high throughput at the cost of minimal lifetime.

Void avoidance depth based algorithms consider both parameters; energy and reliability. However, there exists tradeoff in the form of high latency and high energy consumption because when void hole occurs, an alternative route is discovered by broadcasting the beacon messages among the network nodes. In addition, if the alternative route is not discovered then depth of the source node is adjusted and again the configuration is performed which leads to unnecessary energy consumption and degrades the network performance.

### 2.5. How Our Work is Different from Baseline Scheme?

In this paper, similar to LMPC routing protocol, both proposed schemes (FLMPC-One and FLMPC-Two) also direct multiple copies of a data packet. However, they are different from LMPC as follows:

- LMPC establishes binary tree from the source node. While, FLMPC-One and FLMPC-Two establish binary tree only from the sensor nodes which reside near the layer. It results in energy efficiency.
- LMPC forwards the data packet to the sensor node which lies in the transmission range irrespective of considering void holes. However, both proposed schemes direct data packet on the path which ensures two or three hops neighbor information. It increases PRR of the network.
- In LMPC, data packets not necessarily pass through the sensor nodes residing near the layer. In this way, less copies of data packet are generated from the sparse regions. However, every data packet passes through sensor nodes which lie near the layers to generate multiple copies in both FLMPC-One and FLMPC-Two. This results in reliability and almost elimination of retransmissions.

## 3. FLMPC: Proposed Protocols

In this section, we first describe the network architectures of existing and proposed schemes. As network architecture of both FLMPC-One and FLMPC-Two is the same, therefore, we use term FLMPC to generalize while describing the architecture. Then, we explain propagation model of underwater acoustic signals. Later, we propose two schemes; FLMPC-One and FLMPC-Two. FLMPC-One makes routing decision using information of two hop neighbors. Whereas, FLMPC-Two forwards the data packet after making sure that the forwarder node has neighbor information up to three hops. Both schemes increase reliability of transmission and reduce the chance of data packet entrance in void hole region. Moreover, the energy consumption is reduced by generating binary tree from the node exists at the closest layer of the source node.

### 3.1. Network Architecture

Before describing architecture, few terms need to be defined here: *Relay nodes* are the sensor nodes which not only forward their sensed data but also forward data of higher depth nodes. While, *Cross nodes* are the sensor nodes lying near or at the layers.

**Figure 1:**
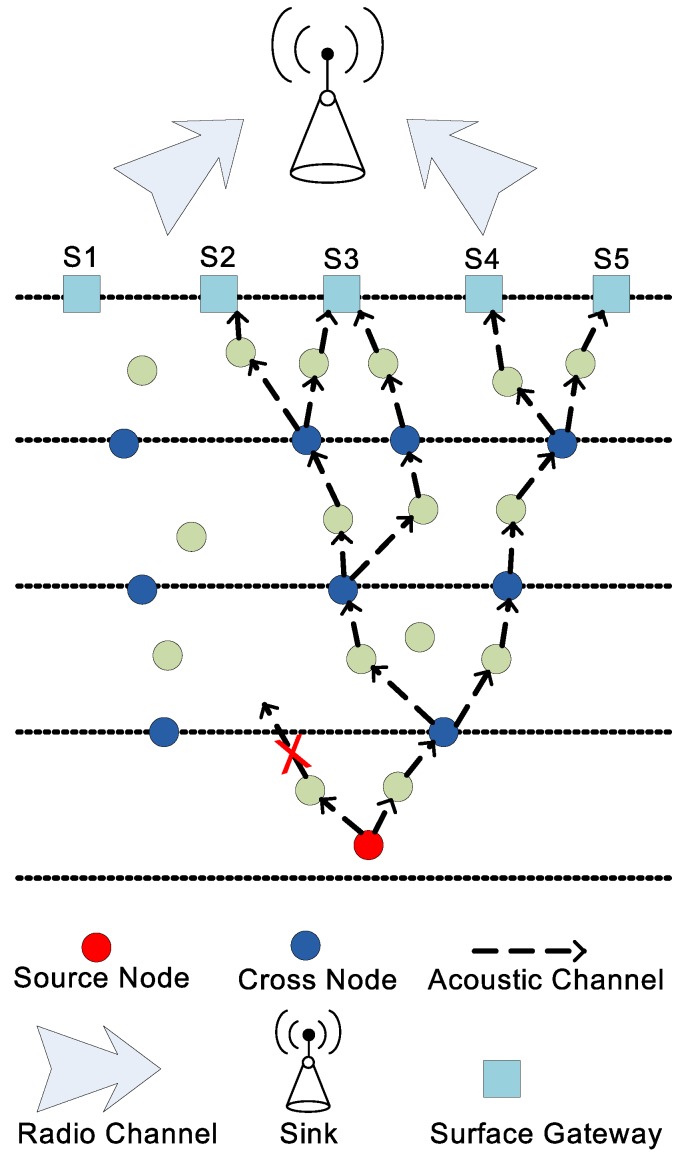
Network Architecture of layered multipath power control (LMPC).

Both LMPC and FLMPC consist of relay nodes, cross nodes, surface gateways and a sink. The sink works as an embedded system to manage the node battery in an efficient way. All the sensor nodes are randomly distributed in the network. Initially, all the nodes are sleeping and become active when they receive data packet. If they are not taking part in communication, they get back to sleep mode. Binary tree is established in both schemes for multiple-path transmission of a data packet. Let’s assume a scenario depicted in Figure 1 in which LMPC network field be distributed into homogeneous layers. From every node, two copies of the data packet are generated irrespective of the layer, until it reaches the destination. Now, it is obvious that noise at the bottom of the sea is very low, where we can afford to generate one data packet, however, the noise increases as we approach the surface of the water.

**Figure 2:**
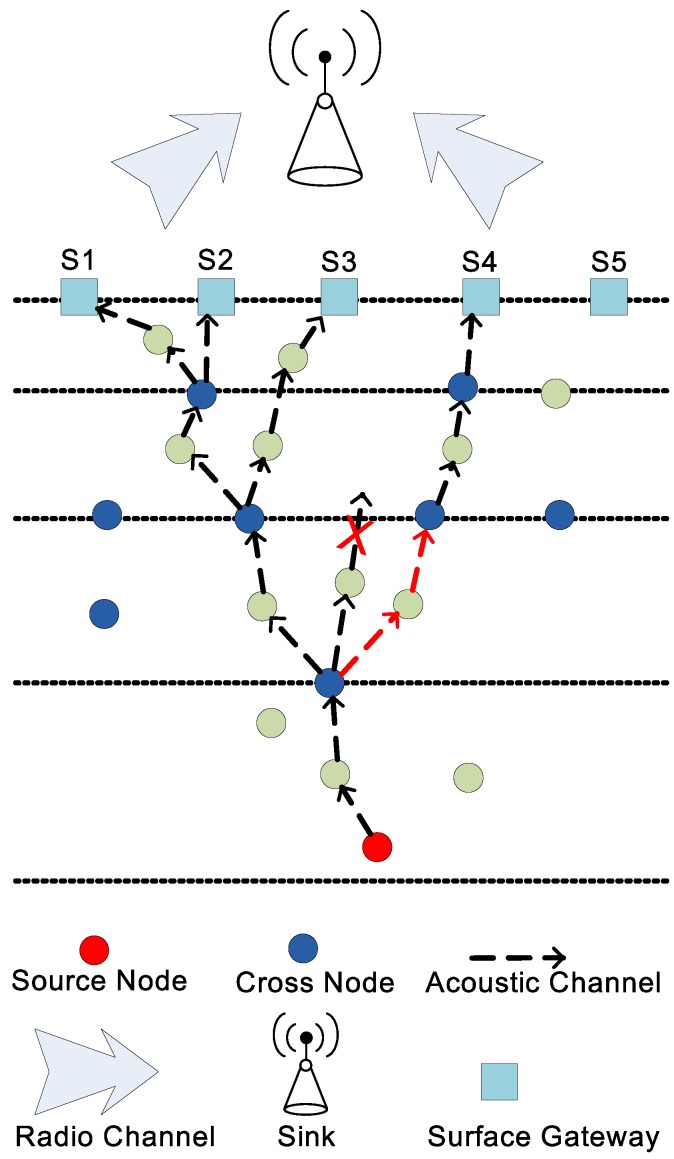
Network Architecture of forward layered multipath power control (FLMPC)-One and FLMPC-Two.

Therefore, we need to generate multiple copies closer to the water surface. In this regard, we have divided the network field into unequal layers as illustrated in Figure 2. In addition, the source node generates only one copy of the data packet at the lower layers, however, as it propagates towards the destination, it starts transmitting two copies through cross nodes deployed over the layer. If, cross node is not present, then only single copy is passed on through a relay node. The source and relay node unicast data packet upon reception. Contrary to source node, a cross node uses IP multicast technology to transmit data packet. Both existing and proposed schemes direct multiple copies of a data packet towards gateways through different paths in order to reduce the retransmissions. All the copies of a data packet are relayed to the sink where they are combined to generate original data packet. Unlike LMPC, FLMPC generates binary tree from cross nodes only with the aim of reducing collisions. Acoustic modem is installed in gateways to receive data from sensor nodes while a radio modem is used to direct collected data towards the sink.

### 3.2. Propagation Model

In this section, we present the propagation model of acoustic signals and their absorption in underwater environment.

#### Energy Model

The attenuation of acoustic channel over distance *d*, for the signal of frequency *f* can be expressed as [20]:

(1) $$A\left(d,f\right)=d^{k}\alpha {\left(f\right)}^{d}$$

where *k* shows the spreading factor of the signal and α(f) is the absorption coefficient. *k* defines the geometry of propagation (i.e., *k* = 1 is cylindrical spreading (signal does not propagate in all directions, but gets trapped within the boundaries and wave fronts move in cylindrical shape without any attenuation in the power of the signal), *k* = 1.5 is practical spreading (spreading loss in which transmission loss and attenuation are considered while performing experiments in order to achieve more accurate and precise results.) and *k* = 2 is spherical spreading (signal propagates in all directions, where wave fronts grow as it move away from the source and intensity of the signal remains the same)). Thorp’s propagation model is used to describe α(f) given by [7]:

(2) $$\alpha \left(f\right)=\frac{0.11f^{2}}{1+f^{2}}+\frac{44f^{2}}{4100+f^{2}}+2.75\times 10^{-4}f^{2}+0.003$$

where α(f) is measured in dB/Km and *f* is given in KHz.

**Figure 3:**
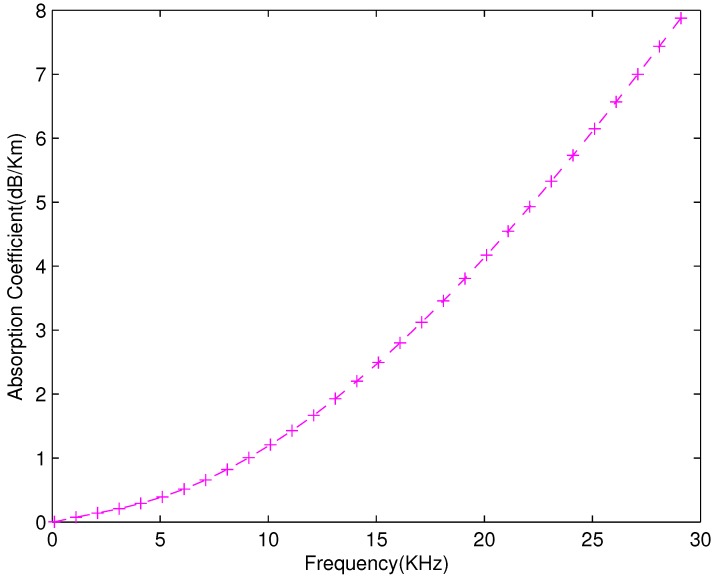
Absorption and Frequency Relation.

Figure 3 shows the relation of α(f) and frequency which is obtained from Thorp’s model. As the frequency increases, absorption of acoustic signals also increases.

The total noise power spectral density (*NP*) of all noises at frequency *f* is given by [5]:

(3) $$NP\left(f\right)=\sum_{i=1}^{ns}NP_{i}\left(f\right)$$

where ns shows the total number of noise sources like shipping, turbulence, etc. and *NP* is measured in dB. The more noise signals propagate away from the source, the more they attenuate. Their attenuation over distance *d* can be calculated from:

(4) $$NP^{d}\left(f\right)=\sum_{i=1}^{ns}\frac{NP_{i}}{d^{k}\alpha {\left(f\right)}^{d}}$$

The signal to noise ratio (*SNR*) of the received signal can be written as:

(5) $$SNR=\frac{P/A(d,f)}{NP^{d}\left(f\right)}=\frac{P}{d^{k}\alpha {\left(f\right)}^{d}\sum_{i=1}^{ns}\frac{NP_{i}}{d^{k}\alpha {\left(f\right)}^{d}}}$$

where *P* is the power of acoustic signal and *d* is the distance among n−1 and *n*th nodes. SNR follows additive white gaussian noise channel (AWGN). As SNR depends on channel modulation scheme through which we can calculate BER. The BER for (π/4) QPSK modulation scheme is given by:

(6) $$p^{ber}\left(SNR\right)=\frac{1}{\sqrt{2\pi }}\int_{SNR}^{\infty }e^{-t^{2}/2}dt$$

BER depends on channel coding scheme and it can be converted to (PER) which is given by:

(7) $$P^{per}=1-{\left[1-p^{ber}\left(SNR\right)\right]}^{Pl}$$

where Pl is the data packet size.

Energy consumption of a sensor node while sending a data packet can be calculated from the values of transmission power (Tp), Pl, data rate (DR) of acoustic channel and *d* is the distance between n−1 and *n*th node. The mathematical expression is given as follows [5]:

(8) $$E_{tx}=\frac{Tp\times Pl}{DR}\times d$$

While, reception energy $\left(E_{rx}\right)$ is calculated according to Equation (9).

(9) $$E_{rx}=\frac{P_{r}\times Pl}{DR}\times d$$

where $P_{r}$ is the reception power. Total leftover energy of the network nodes after forwarding all the data packets is computed as follows:

(10) $$E_{tx}^{rem}=\sum_{dp=1}^{N}\sum_{h=1}^{m}n_{i}-E_{tx}$$

where *N* shows the total number of data packets, *m* is the total hop count for one data packet from the source node to the destination, $n_{i}$ is the *i*th node of the network. When the hop count increases by one, it updates $n_{i}$. We can find the total remaining energy of the network for receiving data packets from:

(11) $$E_{rx}^{rem}=\sum_{dp=1}^{N}\sum_{h=1}^{m}n_{i}-E_{rx}$$

Total consumed energy $E_{total}$ of the network is computed by Equation (12).

(12) $$E_{total}=E_{i}-\left(E_{tx}^{rem}+E_{rx}^{rem}\right)$$

where $E_{i}$ is the total initial energy of the network.

### 3.3. Problem Description

**Figure 4:**
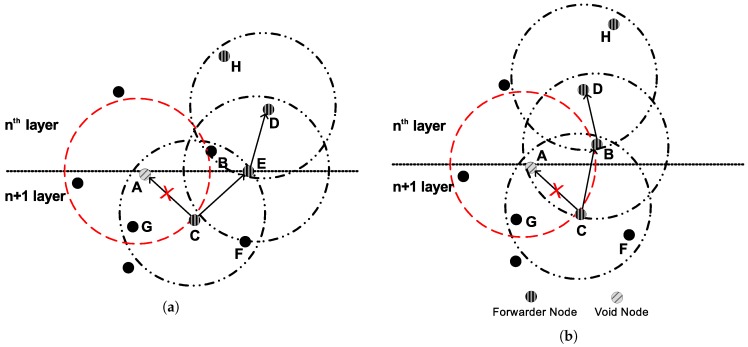
Forwarder Node Selection in FLMPC-One. (**a**) Cross Node potential forwarder node (PFN); (**b**) Relay Node PFN.

The intensity of noise signals is high in shallow water as compared to deep water [21]. In LMPC, the source node establishes binary tree as shown in Figure 1. This establishment increases the number of duplicate packets in dense network and results in collision. Random distribution and energy depletion of sensor nodes create void holes. LMPC forwards data packet on the link which has minimum PER and acceptable DR in the presence of errors without considering the probability of void hole occurrence. As shown in Figure 4a, node *C* is a current forwarder. *A* and *E* are low depth nodes and lies within the transmission range of *C*. Link to *A* might have less errors than *E* and is suitable to become forwarder, however, *A* leads to void hole because it has no node in its transmission range. If *C* directs data packet to *E*, error chances might be higher. However, the probability of the data packet to reach the destination is very high in this situation. LMPC divides the network filed into equal sized layers irrespective of considering noise intensity in shallow water. High noise makes the data packets erroneous which effects the reliability of the data packet.

### 3.4. Layers Division

**Figure 5:**
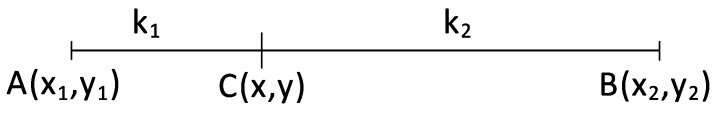
Layer Division.

In FLMPC-One and FLMPC-Two, we split the network into unequal sized layers, because noise intensity is relatively high near the noise source and in the shallow water, while, smoother in deep water. Based upon this fact, the layer size near the surface is smaller in contrast to deep water. This division increases the reliability and reduces the packet drop of FLMPC-One and FLMPC-Two. The expression for unequal size division is given by [22]:

(13) $$C\left(x,y\right)=\left(\frac{k_{1}x_{2}+k_{2}x_{1}}{k_{1}+k_{2}},\frac{k_{1}y_{2}+k_{2}y_{1}}{k_{1}+k_{2}}\right)$$

where $x_{1}$, $y_{1}$, $x_{2}$ and $y_{2}$ are the coordinates of end points and C(x,y) is the point of division as shown in Figure 5. Point *C* divides AB in the ratio $k_{1}$:$k_{2}$ which depends upon the noise signal’s strength.

Cross nodes reside near the layers as shown in Figure 2. When sensor nodes are randomly deployed, they often leave layers vacant. To make sure every data packet passes through cross node, the deployment of cross nodes is uniform random.

### 3.5. Neighbor Table

Sensor nodes reside in transmission range and lying at the low depth than the current forwarder are its neighbors. In order to save energy because of excessive exchange of control messages among the network nodes, all the sensor nodes in proposed schemes maintain neighbor table. Initially, the sensor nodes broadcast control message in the network. This control message consists of source ID, location (*x*,*y*) and depth of source node. Receivers calculate their distance with source based upon receiving a control message by Euclidean distance formula which is given by:

(14) $$Dist_{\left(i,j\right)}=\sqrt{{\left(x_{i}-x_{j}\right)}^{2}+{\left(y_{i}-y_{j}\right)}^{2}}$$

where $\left(x_{i},y_{i}\right)$ and $\left(x_{j},y_{j}\right)$ are the coordinates of source and receiver, respectively.

Every sensor node maintains tuple which consists of (Source ID, Location, Distance, Depth) for each neighbor. The Location is (*x*,*y*) coordinates of the sender retrieved from the control message, whereas, Distance is calculated using Equation (14), Depth shows that how far the node is from the surface of the water. When this information is acquired successfully, the sensor node maintains it’s tuple of neighbor nodes.

(15) $$Dist_{\left(i,j\right)}\leq R_{t}$$

(16) $$Depth_{i}>Depth_{j}$$

where $R_{t}$ is the transmission range and Equation (15) ensures that the calculated distance must be less than or equal to the transmission radius. Receiver nodes also retrieve depth information from the control message to compare it with its own depth as shown in Equation (16). If Equation (16) evaluates to true, the receiver maintains tuple for the source node in its neighbor table. Depth of neighbors helps the sensor nodes to select forwarder which is at minimum depth among all selected neighbors while transmitting data packet. Both schemes do not maintain information of high depth nodes because they have no mechanism of backward transmissions and also to reduce the message overhead.

### 3.6. Network Configuration and Data Transmission

Transmission phase consists of: potential neighbor selection and tree establishment. In the former, a node which is deployed closer to the destination than the source node will be illegible to become a neighbor. Whereas, in latter, tree is established to successfully deliver the data packet from multiple routes towards the destination. We have discussed both of the phases in detail as follows:

#### 3.6.1. Potential Neighbor Selection

When a sensor node has a data packet to transmit, it selects a potential forwarder node (PFN) among all of its neighbors. Before describing the selection process of PFN, we firstly define the term PFN here:

*PFN:* Let a sensor node *n* lying *h* hops away from the destination having a neighbor of h−1 hops which further has neighbors up to h−1 or h−2 hops, this neighbor is a PFN. Sensor node *E* in Figure 4a is a PFN.

In the PFN selection phase, every senor node processes all the neighbors to select cross node which is also a PFN. Cross node increases duplicate packets which helps in achieving the purpose of high reliability. If no such neighbor is found, sensor node selects relay node which is a PFN. In the case that no PFN is found, data packet is dropped to save energy of the node.

##### PFN Selection in FLMPC-One

Figure 4a,b illustrate PFN selection mechanism of FLMPC-One. Let *C* be the current forwarder lying at depth *d* and *h* hops away from the surface of the water. *A*, *B* and *E* lie at h−1 hops. *B* is at lowest depth among all the neighbors, data packet should be sent to it. However, *A* and *E* are cross nodes, they have higher priority than *B*. Existence of cross nodes reduce the chances of data packet transmission to relay node *B*. If we look precisely at Figure 4a, *A* can not be selected as PFN. Although, there is a sensor node at lower depth than *A*. However, it does not lie in A’s transmission range. At this stage, *C* processes only left cross node *E* which has *D* in its range. *D* ensures neighbor information up to h−2 hops. Thus, *E* is a PFN and data packet is transmitted to it.

There can be a scenario, when cross node is not the PFN as shown in Figure 4b. *A* is a cross node having high priority, however, its not a PFN. Now *C* looks in it’s neighbor table to find another cross node. If there is not any cross node then *C* makes an effort to find relay node which is also a PFN. If we look at *B*, we can see, it is a relay node and also has a node in its transmission range, so *B* has priority in this case. *C* selects *B* as a forwarder.
**Algorithm 1** FLMPC Pseudocode.1:**procedure** Potential–Forwarder2:  **for** each node *i* ∈ Nodes **do**3:    **for** each node *n* ∈ Neighbor–Table **do**4:     **if**
$\left|neighbor(n)\right|\geq 1$
**then**5:     **go to** 76:     **if**
$\left|neighbor(neighbor(n))\right|\geq 1$
**then**7:       **if** n==Cross–node **then**8:         PFN←n9:         **go to** 2              ▹ Find PFN for next node10:       **end if**11:     **end if**12:     **end if**13:     **if**
$\left|Cross\overset{◡}{\;}node\right|==0$
**then**14:     **go to** 1615:     **if**
$\left|neighbor(neighbor(n))\right|\geq 1$
**then**16:       PFN←relay17:     **end if**18:     **end if**19:    **end for**20  **end for**21:**end procedure**22:**procedure** Data–transmission23:  Node *i* received data packet24:  **if**
*i* is Cross–node **then**25:    Multicast data packet26:  **else** Unicast data packet27:  **end if**28:**end procedure**


Algorithm 1 shows the selection and transmission of both proposed schemes. Line 4 states that two hop information is fulfilled, thus, line 6 is skipped for FLMPC-One. After PFN selection at step 8, the next iteration starts. Afterwards, transmission phase starts. If current forwarder is a cross node, data packet is multicasted, otherwise, unicasted.

##### PFN Selection in FLMPC-Two

**Figure 6:**
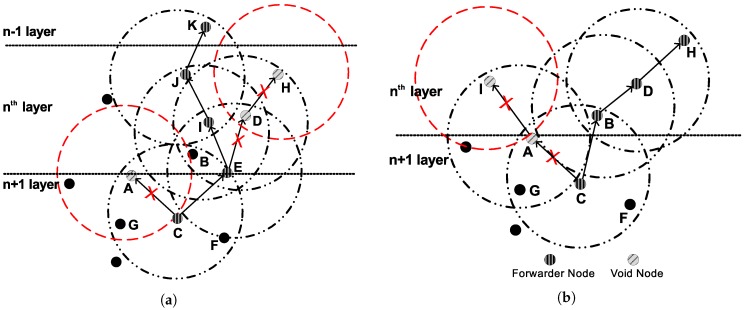
Forwarder Node Selection in FLMPC-Two. (**a**) Cross Node PFN; (**b**) Relay Node PFN.

FLMPC-Two forwards data packets after ensuring neighbor information up to h−3 number of hops from the current forwarder. The PFN selection mechanism of FLMPC-Two is shown in Figure 6a,b. *A*, *B* and *E* are h−1 hops away from the gateways and they are neighbors of the current forwarder *C* which itself is *h* hops away. Let *C* has a data packet to forward and it computes all the neighbors to find PFN. Being cross nodes *A* and *E* has high priority. *A* does not have any node in its range, so *A* is eliminated from this competition. *C* now makes an attempt to spot cross node which is *E* in this state. By focusing on *E* in Figure 6a, we can see, it has *D* and *I* in range which confirms h−2 hops information with respect to *C*. However, its not enough for avoiding the energy hole occurrence in the network. Both *D* and *I* are computed to ensure that there is no void hole. *D* and *I* has *H* and *J* in their transmission ranges, respectively, and based upon them *C* forwards data packet to *E*.

Now, *E* is the current forwarder and also a cross node, so data packet needs to be multicasted. *E* follows complete selection mechanism and spot two relay nodes *D* and *I*. *D* further has *H* in its range. However, *D* can not be the PFN because *E* has only h−2 hops information in case of *D*. *D* is leading to void region and because of no backward transmission, *H* has only choice of packet drop. Node *J* lies in *I*’s transmission range and *K* resides in range of *J*. h−3 hops information is confirmed and *E* now forwards data packet to *I*. Being cross node *E* needs to multicast data packet, however, only one PFN can ensure reliable transmission. FLMPC-One and FLMPC-Two make energy efficient decisions.

If there is no cross node in transmission range or the existing cross node is leading to a void hole, data packet transmission to such node results in packet drop. As shown in Figure 6b, *A* is cross node and it has *I* in range. However, *A* leads the data packet to a void hole. Now, *C* looks in the neighbor table to locate another cross node. In the case shown in Figure 6b, this search results in negation. *C* now searches for relay nodes and spots *B* which ensures h−3 hops information. The data packet is forwarded to PFN*B*.

#### 3.6.2. Tree Establishment

When a sensor node sends a data packet, one of the given conditions is true. The current forwarder is a cross node, a source or a relay node.

(17) $$Y=\left\{\begin{array}{cc} L; & \mathrm{if}\;N=S_{n}\;\mathrm{or}\;N=N_{n}\\ T; & \mathrm{if}\;N=C_{n} \end{array}\right.$$

where *L*, *T*, *N*, $S_{n}$, $N_{n}$ and $C_{n}$ represent the linear transmission, tree, current forwarder, source node, relay node and cross node, respectively.

If *N* is $S_{n}$ or $N_{n}$, it unicasts the data packet and follows a linear path. However, incase *N* is $C_{n}$, it multicasts data packet and generates binary tree. A tree consists of a root node and subtrees. In case of a binary tree, every parent node has utmost two subtrees. In our schemes, only $C_{n}$ generates binary tree. The establishment of binary tree can be expressed as [5]:

(18) $$T=G(B_{1},P_{0},B_{2})$$

where

(19) $$B_{i}=\left\{\begin{array}{c} G(B_{2i+1},P_{i},B_{2i+2});\\ \;\;\;\;\;\;\mathrm{if}\;B_{2i+1}\neq \phi \;\mathrm{and}\;B_{2i+2}\neq \phi \\ P_{i};\\ \;\;\;\;\;\;\mathrm{if}\;B_{2i+1}=\phi \;\mathrm{and}\;B_{2i+2}=\phi \end{array}\right.$$

where, the ϕ is used to represent the leaf node. If, $B_{2i+1}$ equals to ϕ, it means no further nodes are present to traverse. If, $B_{2i+1}$ not equals to ϕ, then the current node is not a leaf node. $P_{0}$ in Equation (18) is the root node, $B_{1}$ and $B_{2}$ are the left and right children which can be leaf nodes or trees. If $B_{1}$ or $B_{2}$ does not have children (low depth nodes), they are leaf nodes. In existing and proposed schemes, leaf nodes are the surface gateways as shown in Figure 1. $P_{i}$ in Equation (19) is the parent of *i*th subtree. Left and right children of *i*th subtree can be found from $B_{2i+1}$ and $B_{2i+2}$.

## 4. Linear Optimization

*Linear Optimization* is a mathematical optimization model widely used to gain the best possible result. It consists of an objective function in which we formulate problem with the aim of minimizing or maximizing the result under a set of linear constraints.

In this section, two linear problems are formulated, the first one is to minimize the energy consumption and the second one is to maximize the throughput of the network.

### 4.1. Energy Minimization

In FLMPC-One and FLMPC-Two, cross nodes multicast and relay nodes unicast data packet. In order to increase the lifetime of network, efficient energy consumption is necessary. Energy is mainly consumed by transmission or reception of data packets. Energy is also consumed in idle state, however, it is very minor, thus, we are not considering it. The objective function of energy can be expressed as:

(20) $$Min\sum_{t=1}^{t_{max}}E\left(t\right)\;\;\forall \;t\;\in \;R$$

such that;

(21a) $$\begin{array}{c} L_{1}:E_{t}\leq E_{o} \end{array}$$

(21b) $$\begin{array}{c} L_{2}:DR\leq DR_{pr} \end{array}$$

(21c) $$\begin{array}{c} L_{3}:E_{s}\geq E_{tx} \end{array}$$

(21d) $$\begin{array}{c} L_{4}:LQ_{t}\geq LQ_{req} \end{array}$$

(21e) $$\begin{array}{c} L_{5}:Min\sum_{dp=1}^{N}E_{tx}\;\forall \;i\;\in \;R \end{array}$$

(21f) $$\begin{array}{c} L_{6}:Min\sum_{dp=1}^{N}E_{rx}\;\forall \;i\;\in \;R \end{array}$$

The main objective of Equation (20) is to minimize the energy consumption for the total time ($t_{max}$) of transmission to prolong network lifetime. Equation (21a) shows that energy of sensor node at any time *t* must be less than or equal to the initial energy ($E_{o}$) supplied to the sensor node. It reveals sensor nodes have limited energy. In Equation (21b), DR must be less than or equal to DR which makes the data packet erroneous. If Equation (21b) evaluates to false then PER increases which results in packet drop. As a result, the data packet needs to be retransmitted and it increases energy consumption. Equation (21c) shows sensor node must have energy greater or equal to transmission energy when it has a data packet to send. Constraint in Equation (21d) ensures that the link quality at current time must be good. If it is not, data packet becomes erroneous and gets dropped at surface gateway. Equations (21e) and (21f) indicate the energy consumption for the total number of data packets $\left(dp_{s}\right)$ transmitted and received in the network. We can calculate $E_{tx}$ from Equation (8) and $E_{rx}$ from Equation (9).

**Graphical Analysis:** Let us consider a scenario in which initial energy be 6 J, Tp = 0.66 mW (milliWatt), Pl = 500 bytes, DR = 10 KB/s, *d* = 100 m to 300 m and Rp = 0.395 mW. From *d* = 100 m to 300 m, the calculated value of $E_{tx}$ is 3.3 mJ to 9.9 mJ and $E_{rx}$ is 1.98 mJ to 5.93 mJ. So

(22) $$3.3\;\mathrm{mJ}\leq E_{tx}\leq 9.9\;\mathrm{mJ}$$

(23) $$1.98\;\mathrm{mJ}\leq E_{rx}\leq 5.93\;\mathrm{mJ}$$

**Figure 7:**
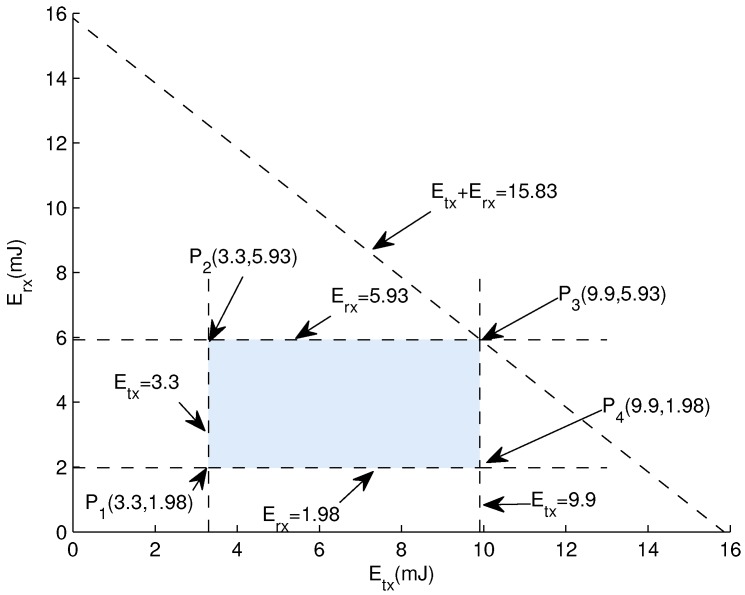
Energy Consumption Feasible Region.

Figure 7 shows the intersection of lines which results in the bounded region which is called feasible region. Every point in this region results in feasible solution. Extreme values can be found at the corner points. Now examining vertices of the bounded region of Figure 7 as follows:

- $P_{1}\left(3.3,1.98\right)$ = 3.3 + 1.98 = 5.28 mJ
- $P_{2}\left(3.3,5.93\right)$ = 3.3 + 5.93 = 9.23 mJ
- $P_{3}\left(9.9,5.93\right)$ = 9.9 + 5.93 = 15.83 mJ
- $P_{4}\left(9.9,1.98\right)$ = 9.9 + 1.98 = 11.88 mJ

The above results show that values of the corner points lies within the range of initial energy. So all solutions are valid. When a sensor node transmits or receives a data packet, the value of energy is selected from this feasible region.

### 4.2. Throughput Maximization

In this section, we are using linear programming to maximize network throughput. In FLMPC-One and FLMPC-Two, multiple copies of a data packet are relayed towards the destination. Throughput is the number of distinct packets successfully received at the sink. The objective function is defined as follows:

(24) $$Max\sum_{t=1}^{t_{max}}P\left(t\right)\;\;\forall \;t\;\in \;R$$

where,

(25) $$P\left(t\right)=\sum_{t=1}^{t_{max}}pt\times c\;\;\forall \;c\;\in \;P$$

and,

(26) $$pt=\left\{\begin{array}{c} 1\;\;\;\;\;if\;distinct\;packet\\ 0\;\;\;\;\;if\;duplicate\;packet \end{array}\right.$$

(27a) $$\begin{array}{c} L_{1}:E_{t}\leq E_{o} \end{array}$$

(27b) $$\begin{array}{c} L_{2}:DR\leq DR_{pr} \end{array}$$

(27c) $$\begin{array}{c} L_{3}:E_{s}\geq E_{tx} \end{array}$$

(27d) $$\begin{array}{c} L_{4}:PER<PER_{req} \end{array}$$

(27e) $$\begin{array}{cc} & L_{5}:Min\sum_{d=d_{o}}^{d_{max}}No_{th}\;\forall d\;\in \;R \end{array}$$

(27f) $$\begin{array}{c} L_{6}:Min\sum_{d=d_{o}}^{d_{max}}No_{s}\;\forall d\;\in \;R \end{array}$$

In Equation (24), our objective is to maximize the number of distinct packets *P* received at the sink in aggregated time ($t_{max}$). In Equation (25), pt is the packet type and *c* is the counter. Counter *c* increments by one if pt is 1 in Equation (26). It ensures that the data packet is not a duplicate one. Equations (27a)–(27c) are same as explained in previous section without them data packet can not be transmitted. Constraint Equation (27d) ensures that PER must be less than or equal to required level ($PER_{req}$). Equations (27e) and (27f) show the thermal noise $\left(No_{th}\right)$ and shipping noise $\left(No_{s}\right)$, respectively. Where *d* is the distance from source node to surface gateways. If noise intensity is high, data packet becomes erroneous. If PER increases the required level, data packet is dropped at gateway. In order to increase throughput, noises should be minimum. Total noise is given by:

(28) $$No_{total}=No_{th}+No_{s}\;\;\;\forall \;No_{th},No_{s}\in \;R$$

where $No_{th}$ [23] and $No_{s}$ [24] are given by:

(29) $$No_{th}=\frac{15+20log(f)}{D}$$

(30) $$No_{s}=\frac{NL-20log(\frac{f}{100})}{D}$$

where NL is noise level and it is based upon shipping density.

**Graphical Analysis:** Let *f* = 1000 Hz, *d* = 100 m to 2000 m and NL = 60 dB. The calculated value of $No_{th}$ is 0.0375 dB to 0.75 dB and $No_{s}$ lies in the range of 0.02 dB to 0.4 dB.

(31) $$0.0375+0.02\leq No_{th}+No_{s}\leq 0.75+0.4$$

(32) $$0.0375\leq No_{th}\leq 0.75$$

(33) $$0.02\leq No_{s}\leq 0.4$$

**Figure 8:**
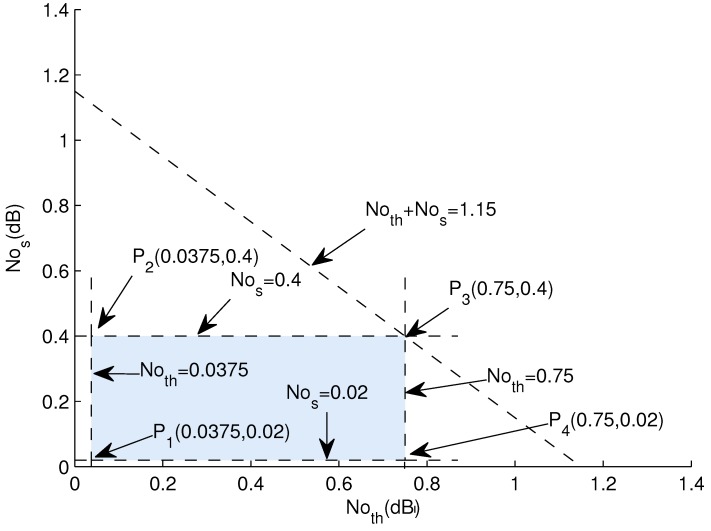
Noises Feasible Region.

In Figure 8, intersection of lines results in feasible region.

All points in this region provide a valid solution. Now testing corner points to validate the results of Equations (32) and (33). Calculating values at corner points:

- $P_{1}\left(0.0375,0.02\right)$ = 0.0375 + 0.02 = 0.0575 dB
- $P_{2}\left(0.0375,0.4\right)$ = 0.0375 + 0.4 = 0.438 dB
- $P_{3}\left(0.75,0.4\right)$ = 0.75 + 0.4 = 1.15 dB
- $P_{4}\left(0.75,0.02\right)$ = 0.75 + 0.02 = 0.77 dB

The lesser the noise is, the higher are the chances of successful packet reception at gateways. If a data packet faces noise value from this feasible region, the error is less in data packet and it is acceptable at gateways.

## 5. Simulation Results and Performance Evaluation

In this section, we evaluate the performance of FLMPC-One and FLMPC-Two. In FLMPC-One, every sensor node looks up to two hop neighbors and finds PFN. While, FLMPC-Two uses three hops neighbor information to forward data packet. The chance for a data packet to enter void hole region reduces. Void hole avoidance results in fewer number of drop packets. In the simulations, we implemented two different scenarios of LMPC, FLMPC-One and FLMPC-Two. In the first scenario, 150 sensor nodes are deployed in the network of 2000 m × 2000 m. We consider two noise sources here i.e., wind and shipping noise. Wind speed is considered 5 m/s and shipping noise is 0.2 dB. Similarly, in scenario two, 500 sensor nodes are distributed over the area of 4000 m × 4000 m. The wind speed and shipping noise are stronger in scenario two i.e., 20 m/s and 0.8 dB, respectively. The transmission power and the reception power of sensor nodes are 0.66 mW and 0.395 mW, respectively. Five gateways are deployed at the surface of water. All surface gateways relay data packets to a sink which combines them to generate original data packet. Simulations are run for 1000 s. The average of five simulation results is taken in order to compensate the difference of random deployment. The network dimensions and important performance parameters for simulations are listed in Table 4.

### 5.1. Performance Metrics

We are defining few terms here based upon them we evaluate proposed and existing schemes.

*Active Nodes* are the number of sensor nodes which are taking part in data transmission and reception.

*Delay* is the time taken by a data packet from the source to the destination. It includes transmission delay, propagation delay, holding time and processing delay. It is measured in second(s). There are multiple surface gateways and more than one copies of the same data packet are relayed, hence a data packet corresponds to more than one delays. However, we consider the highest delay among them.

*Energy Consumption* is the amount of energy consumed from start to the end of the network lifetime. Total energy consumption comes from the accumulation of energy consumed by individual nodes during the data transmission phase.

*Required Packet Error Rate $\left(Pr_{req}\right)$* is the maximum number of erroneous bits allowed in the data packet to be accepted at sink. If erroneous bits in data packet exceeds $Pr_{req}$ limit, surface gateway drops it.

*PRR* is the ratio of successfully received data packets at surface gateways to the data packets sent by source nodes.

### 5.2. Simulation Results

In this section, we have presented and discussed the simulation results for both FLMPC-One and FLMPC-Two. Both schemes are compared with LMPC in terms of active nodes, delay, energy consumption, required packet error rate and PRR. The detailed discussion of each parameter along with it’s results is presented as follows:

#### 5.2.1. Active Nodes

**Figure 9:**
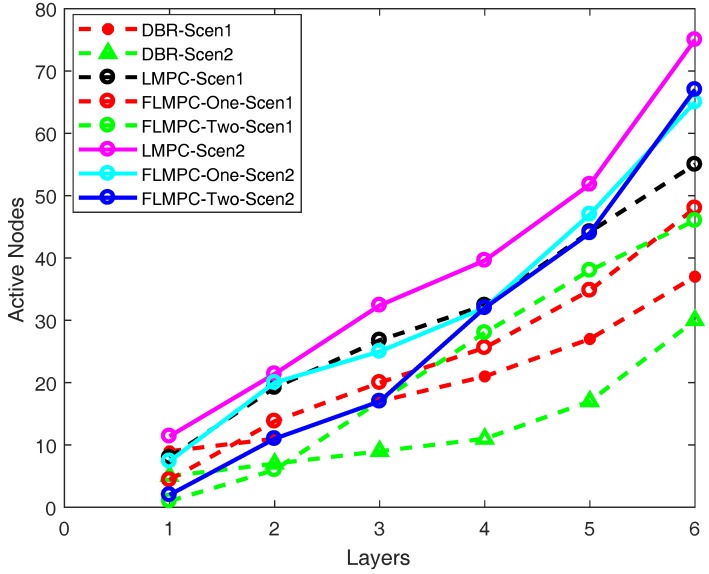
Active Nodes.

It can be observed from Figure 9, as the number of layers increases number of active nodes also increases. Because if the source node lies nearest to surface gateways, it results in less number of duplicate packets and awake less number of nodes. However, if source node resides away from the surface, it results in high number of active nodes. It shows farther the source node is from the surface, higher is the number of active nodes.

The number of active nodes increases as the layer number increases in DBR. When the information is obtained up to two hops, the active nodes are high, however, as the hop count increases, means the neighbor information acquired up till three hops, the probability of finding void hole increases. Furthermore, the transmission of data packet from the single path towards destination only involves few number of nodes. While in LMPC and FLMPC multiple routes generated from every cross node results in high number of active nodes. The use of single route is the major reason behind low number of active nodes participating in the data forwarding process.

Figure 9 shows that initially the number of active nodes in scenario one of LMPC is high due to tree generation from source node resulting in high number of duplicate copies. Later, the increment is gradual because higher depth nodes forward data packets through the relay nodes which are already awake in the upper layers and also awake few more sensor nodes. Less number of active nodes save network energy.

Number of active nodes in FLMPC-One is lower as compared to LMPC. FLMPC-One generates less copies of packets which reduces the chances of collision. Packet collision is very critical factor in sensor networks which effects energy and delay of the network. If collision increases, it affects network’s performance and results in loss of data. All the active nodes not only relay data packets of higher depth nodes but also sense their own data and forward it. Two hops neighbor information of FLMPC-One reduces number of active nodes and also reduces the probability of data packet entrance in void region. FLMPC-One acquires neighbor information to avoid the awakening of sleeping nodes leading to void regions.

Active nodes of FLMPC-Two in scenario one are less than both LMPC and FLMPC-One because of finding PFN after using three hops information. It saves data packet from entering in void region. If sensor node is lying in the sparse region, this mechanism reduces the probability of data packet drop. When a sensor node forwards data packet to its PFN using three hops information. The receiver node needs not to forward that data packet to the node found by its previous forwarder during PFN finding mechanism. Because previous node information may lead data packet to void region. So, current forwarder locates its PFN by running PFN selection mechanism. In this way, data packets loss is avoided.

In scenario two, initially increment in the number of active nodes of LMPC and FLMPC-One is rapid. Later, FLMPC-One makes smart decision by detecting void holes and changes the path of the data packet. In this way, FLMPC-Two awakes suitable nodes which results in gradual increment. Although, two hops neighbor information reduces active nodes of FLMPC-One, however, three hops information reduces them more. FLMPC-Two has more data to forward and already awake nodes are not enough. So, active nodes of FLMPC-Two increases sharply as the time passes on, however, it awakes most suitable sensor nodes.

#### 5.2.2. Delay

**Figure 10:**
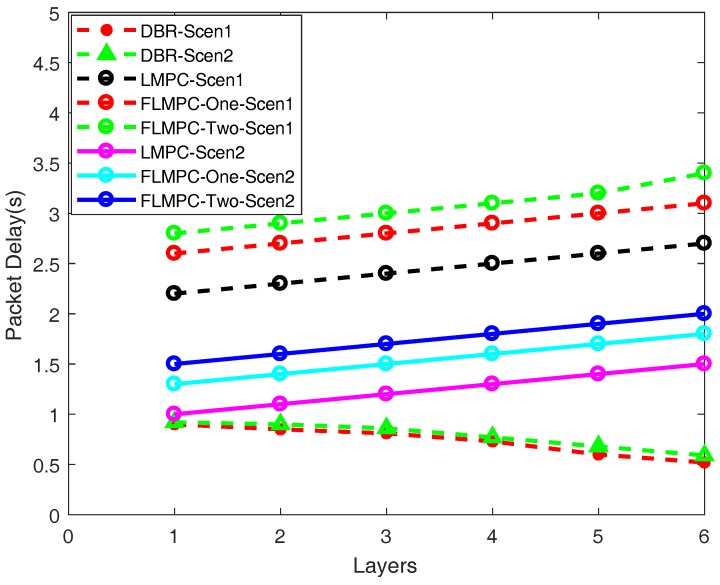
End-to-end Delay.

Delay of proposed and existing schemes is shown in Figure 10 and it mainly depends upon underwater channel and distance from the source to destination. As expected, delay of LMPC in both scenarios is less as compared to FLMPC-One and FLMPC-Two, respectively, because of one hop information which leads to high number of active nodes as shown in Figure 9. As the number of active nodes increases, the probability of finding PFN in less time also increases. Figure 10 shows delay of all schemes in scenario one is higher than scenario two. The difference in sensor nodes density of both scenarios is one of the reasons. Scenario one is 70% less dense as compared to scenario two.

Due to single path routing, the delay is very less in the both scenarios of DBR. The classic depth based algorithm only considers distance while making forwarding decisions. The selection of always shortest path irrespective of energy consumption results in minimal delay. It is evident from the Figure 10, that as number of layers increases, the number of active nodes also increases resulting in further decrease in the end-to-end delay. At layer 6, the minimal delay is 0.51 and 0.53 s in scenario one and scenario two, respectively.

In scenario one, the delay of FLMPC-One is almost 0.2 s higher than LMPC because of processing time for potential node selection at every node. Meanwhile, FLMPC-Two’s delay is almost 0.6 s higher than LMPC due to less number of active nodes and three hops neighbor calculation. Less number of active nodes results in less number of possible paths to advance data packet reliably towards surface which increases processing delay.

In scenario two, delay of FLMPC-One is almost 0.3 s higher and FLMPC-Two is 0.5 s higher as compared to LMPC. The reason to locate PFN using two hops or three hops information is to avoid energy holes. When a data packet reaches in void region, there is no way out and the current forwarder drops it which needs to be retransmitted. Retransmission increases energy consumption and delay.

In existing and proposed schemes, multiple copies of a data packet reach at the destination. So, there are more than one delays for one data packet. In our proposed schemes, we consider the worst scenario and count the highest delay among them. While calculating delay, we are taking into consideration transmission delay, propagation delay and processing delay of PFN at each node. In proposed schemes, if there are more than one, two or three hops neighbors exist then the one farthest among them from current forwarder is selected. The farthest node selection affects delay because of high propagation time.

#### 5.2.3. Energy Consumption

**Figure 11:**
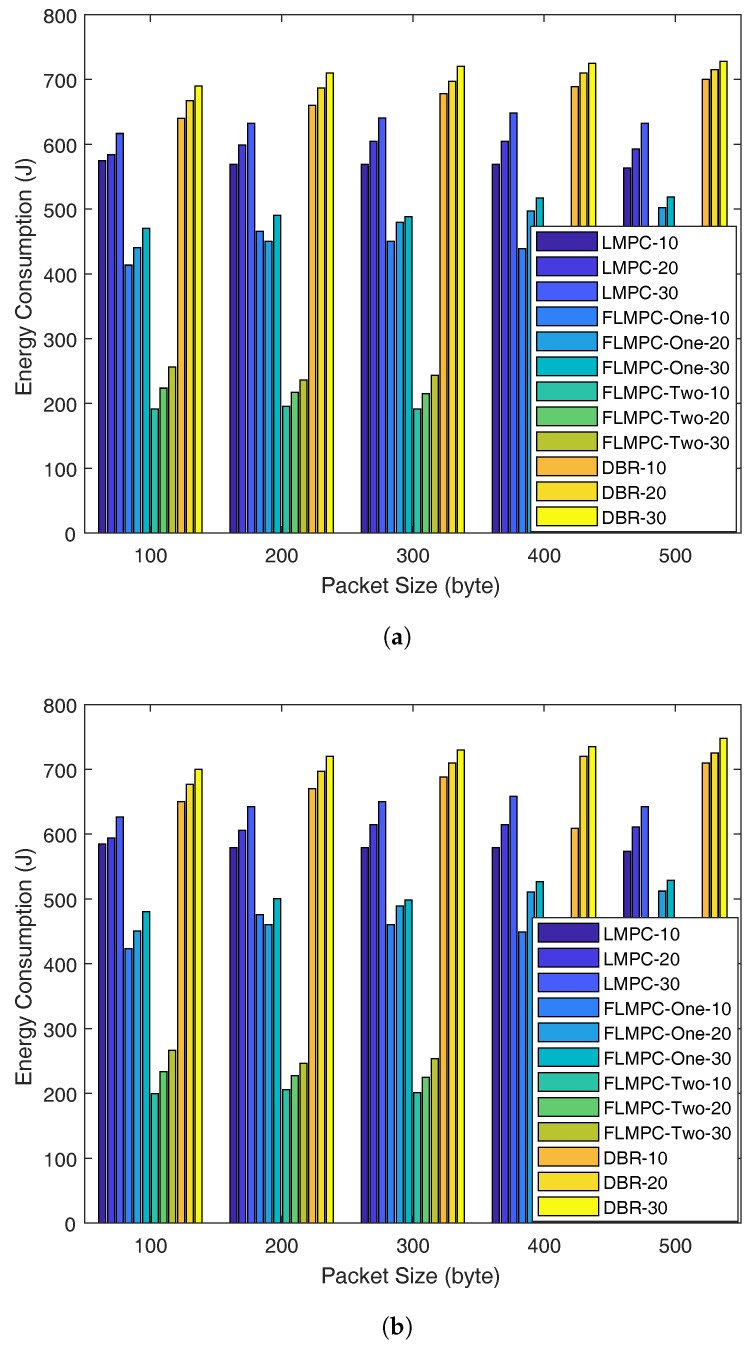
Energy Consumption with Different Data Rate and Packet Size. (**a**) Scenario-1; (**b**) Scenario-2.

Figure 11a,b show energy consumption of existing and proposed schemes in scenario one and scenario two, respectively, with different DRs and Pls. Energy consumption depends upon the DRs irrespective of the Pl. We set DR to 10, 20 and 30 KB/s and Pl to 100, 200, 300, 400 and 500 bytes.

High number of active nodes in LMPC as shown in Figure 9, increases collision in the dense network which results in packet drop. While, sparse regions effect PRR by dropping data packets in void region. In existing and proposed schemes, multiple copies are directed towards surface in order to eliminate retransmissions and save energy.

**Figure 12:**
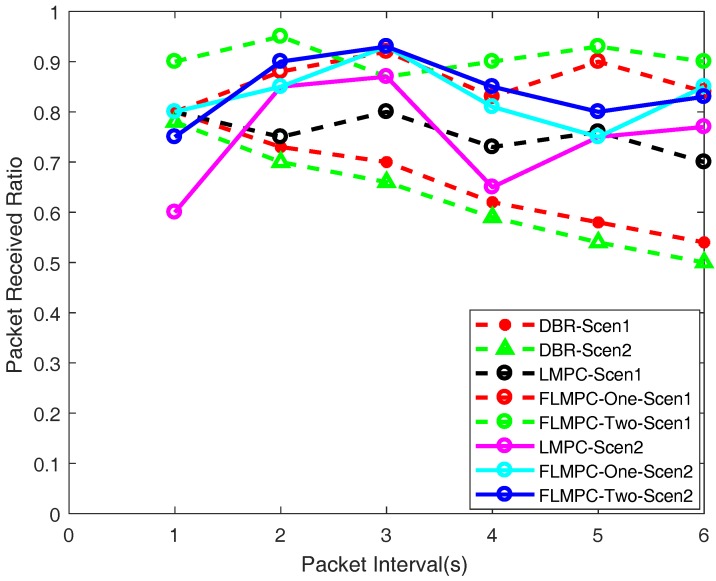
Packet Received Ratio.

Unlike LMPC, tree generation from source node, FLMPC-One and FLMPC-Two establish binary tree from cross nodes only which results in less number of duplicate data packets. FLMPC-One and FLMPC-Two reliably transmit data packets to most suitable nodes and save data packets from entering void region. These reliable and less number of data packets ultimately saves energy and increases PRR as shown in Figure 12.

In scenario one, FLMPC-One saves almost 8–12.5% energy while, FLMPC-Two saves 23–26% energy of the network as shown in Figure 11a. FLMPC-One saves 8.33–12.7% energy and FLMPC-Two saves 23.8–29% energy in scenario two. FLMPC-One and FLMPC-Two reduces energy consumption at the cost of delay. In FLMPC-Two, every node performs calculations up to three hops in order to avoid void holes which increases PRR and saves energy. FLMPC-Two achieves this by paying cost of high delay as shown in Figure 10.

In DBR, energy consumption is higher in both scenarios one and two as compared to LMPC and FLMPC schemes. The high energy consumption is because of void hole occurrence when forwarding data packets with greedy approach. Moreover, it can be seen from Figure 11a,b, that with the increase in packet size, the energy consumption also increases. The reason for more energy dissipation in DBR is because of inefficient approach to handle the void holes. The occurrence of void holes increase the packet drop ratio resulting in unwanted energy depletion. While in LMPC and FLMPC transmission of data packets from multiple paths and generation of data packets from cross nodes ensure that the packet will not be re-transmitted.

#### 5.2.4. PRR

It can be seen from Figure 12 that PRR of LMPC in scenario one is less than in both proposed schemes. Initially, it is higher and it gradually decreases as the time increases. Because establishment of binary tree from source node increases duplicate packets in dense region which results in high collision. This collision increases packet drop. Moreover, one hop information in LMPC may lead data packet to void region which also affects PRR. In scenario two of LMPC, initially PRR is less because of less number of copies reached to gateway. However, it improves as the time increases because of increasing active node density. As active nodes increases, number of possible paths increases.

PRR of DBR in both scenarios is less as compared to both FLMPC schemes and LMPC. The reason for low performance in term of packet reception is greedy approach for at every data packet is broadcasted to find out a shortest path. The broadcast mechanism increases the collision of the data packets. Moreover, with the increase in number of active nodes, the chances of collision also increases. In addition, the factor of holding time also contributes in the reduction of PRR at each hop. Hence, from the depicted results it can be concluded that high collisions and inefficient holding time of the data packet by the neighbors node result in less PRR.

In the proposed schemes, unlike LMPC, every source node unicasts the data packet. When this data packet reaches the the cross node of nearest layer, this cross node generates a binary tree. This mechanism results in a lower number of duplicate packets which reduces collision as compared to LMPC. In a sparse region FLMPC-One’s two hops and FLMPC-Two’s three hops neighbor information avoid void holes. In void holes avoidance, proposed schemes awake most suitable sensor nodes to lead data packet towards destination. There are less copies generated in both proposed schemes as compared to LMPC. However, reliable advancement of data packets increases PRR. PRR of FLMPC-One in scenario one is higher than PRR of scenario two because it has less active nodes in scenario one which ultimately leads to less collision.

#### 5.2.5. Required Packer Error Ratio

**Figure 13:**
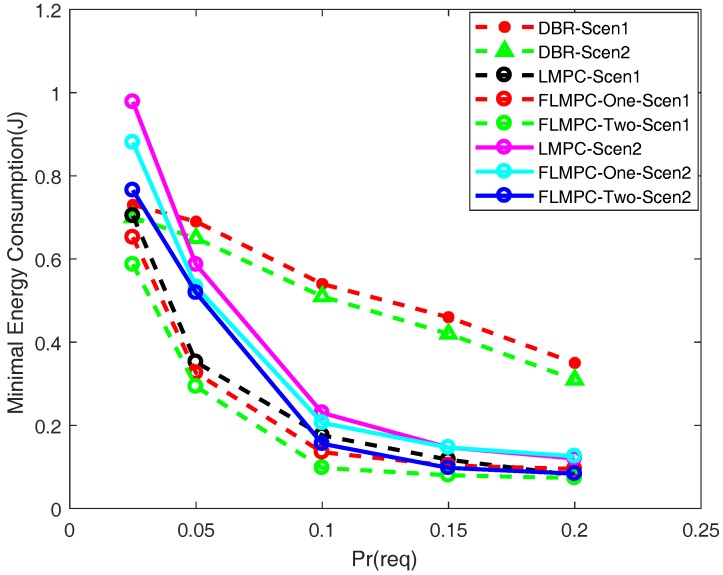
Required Packer Error Ratio.

Figure 13 shows energy consumption per packet in the presence of different $Pr_{req}$. We can observe, if $Pr_{req}$ is high which means high reliability of data packet, energy consumption is also high. Because when data packets are received at the gateway, error is checked in them. If error is higher than threshold, this data packet is dropped. As we allow higher number of acceptable erroneous bits, energy consumption decreases. Increasing $Pr_{req}$ value means that reliability of data packet is decreasing.

Energy per packet with different $Pr_{req}$ in both scenarios of LMPC is higher as compared to proposed schemes. Although, the difference is less because in all schemes multiple copies are generated of a data packet. So, data packets reach at the sink through different paths. If one path has high fading, data packet may get less affected on other path. FLMPC-One is performing better because of two hops information and achieves reliability in both scenarios. Figure 13 shows that FLMPC-Two outperforms both schemes because of three hops information. FLMPC-Two selects that path which advances data packet towards surface and effects the data packet less. If all the copies of the data packet are erroneous then still there is a chance of getting original packet by combining them at the sink.

The energy consumption of DBR in Figure 13 is initially low as compared to LMPC and FLMPC because it only generates one copy from every source node. On the other hand, with the increase in packet interval, the energy depletion increases because of high message exchange among the network nodes. Additionally, the occurrence of void hole also increases which results in high energy dissipation and more packet drop ratio. In Figure 13, the results depicted clearly show that at 6 s of packet interval, the energy consumption is greater than 0.3 J. Hence, the FLMPC and LMPC have achieved considerable amount of improvement in terms of energy utilization as compared to DBR.

### 5.3. Performance Trade Offs

Table 5 shows the performance trade offs of proposed schemes. In order to avoid repetition, parameters are explained generally because of same behaviour in proposed schemes. Multiple copies of data packets are forwarded in order to achieve reliability. FLMPC-One performs better in terms of active nodes, energy consumption and PRR. However, processing delay at each node in FLMPC-One increases its delay. FLMPC-Two makes smart decisions than FLMPC-One and awakes less number of active nodes which saves energy and increases PRR by reliably transmitting the data packet towards the surface. However, FLMPC-Two pays the cost of delay.

## 6. Conclusions

Energy conservation is one of the prime requirements in protocol designing of UWSNs because of limited resources. Energy holes, created by random distribution or death of nodes, increase the data packet’s drop rate and decrease the network lifetime. In this paper, we have proposed FLMPC-One and FLMPC-Two in order to achieve reliability and energy efficiency. Both schemes direct multiple copies of a data through various data routes towards the surface gateways where they are combined to generate an original data packet. Deep water faces less noises; thus, both proposed schemes (FLMPC-One and FLMPC-Two) have not generated the binary tree from cross nodes deployed in deep water. This is only done to reduce number of duplicate packets at the destination in order to save nodes battery and prolong the network operations. However, in shallow water, the noise increases, therefore cross nodes generate binary tree and direct multiple copies towards the destination which ensured the reliable delivery of data packets. FLMPC-One and FLMPC-Two avoid the void hole problem through a proactive approach and find an alternative route towards the destination making sure that the selected path has less noises. The simulation results show that our proposed schemes achieve energy efficiency, high PRR and reduce the number of active nodes which increases the network lifetime by paying the cost of delay.

## Figures and Tables

**Table 1:** Comparison of Single Path Routing Protocols.

Protocol Name	Features	Achievements	Limitations
DBR [6]	Multi-hopping	High PDR and less delay	Void holes waste energy and increase retransmissions
WDFAD-DBR [7]	Multi-hopping	Increases PDR and energy efficiency	Delay of network increases
R-ER$P^{2}$R [8]	Multi-hopping	Maximize network lifetime and balanced energy consumption	Void regions increase delay
VAPR [9]	Multi-hopping using depth information	Energy efficiency	High delay and low PDR in sparse regions
D-DBR [10]	Multi-hopping	Less delay and high PDR	High energy consumption
GFGD [11]	Multi-hopping among small cubes	Energy efficiency and less delay	No mechanism to avoid void holes

**Table 2:** Comparison of Multi-Path Routing Protocols.

Protocol Name	Features	Achievements	Limitations
MPT [12]	Multiple path transmission	Increases reliability and low delay	Dense regions increase collisions and energy consumption
AEDG [13]	AUVs and multi-hopping	Balanced energy consumption and high throughput	High delay
PER [14]	Forwarding data through multiple paths by following binary tree	High PDR	High delay and energy consumption
M-FEC [15]	Data forwarding through multiple paths	Minimum energy consumption and reliability	Delay increases because of data packet correction at each node
EBH [16]	Direct transmission and multi-hopping	Balance energy consumption	Void holes increase delay and energy consumption

**Table 3:** Comparison of Void Avoidance Routing Protocols.

Protocol Name	Features	Achievements	Limitations
GEDAR [17]	Multi-hopping	Void holes coverage	High energy consumption due to nodes movement
HYDROCAST [18]	Multi-hopping through multiple paths	Reliable transmission and PDR	High delay and energy consumption
SHORT [19]	Multi-hopping	High throughput and extended network lifetime	High delay

**Table 4:** Simulation Parameters.

Option	Value	
	Scenario-1	Scenario-2
Area	2000 m × 2000 m	4000 m × 4000 m
Noise of Ship	0.2 dB	0.8 dB
Wind	5 m/s	20 m/s
Number of Nodes	150	500
Simulation Period	1000 s	
Frequency	914 × 10^6^ Hz	
Initialized Energy	1000 J	
txPower	0.66 mW	
rxPower	0.395 mW	
idlePower	0.035 mW	

**Table 5:** Performance Tradeoffs in Proposed and Compared Schemes.

Protocol	Achievements	Figure	Compromised Parameter	Figure
LMPC	Reliability and delay	Figure 10	Energy consumption	Figure 11a,b
			active nodes and PRR	Figure 9, Figure 12
FLMPC-One	Reliability, energy consumption	Figure 11a,b	Delay	Figure 10
	active nodes and PRR	Figure 9 Figure 12		
FLMPC-Two	Reliability, energy consumption,	Figure 11a,b,	Delay	Figure 10
	active nodes and PRR	Figure 9, Figure 12		
